# Analysis of endogenous and exogenous tumor upregulated promoter expression in canine tumors

**DOI:** 10.1371/journal.pone.0240807

**Published:** 2020-11-09

**Authors:** Abdul Mohin Sajib, Maninder Sandey, Samantha Morici, Bradley Schuler, Payal Agarwal, Bruce F. Smith

**Affiliations:** 1 Scott-Ritchey Research Center, College of Veterinary Medicine, Auburn University, Auburn, AL, United States of America; 2 Department of Pathobiology, College of Veterinary Medicine, Auburn University, Auburn, AL, United States of America; Sechenov First Medical University, RUSSIAN FEDERATION

## Abstract

Gene therapy is a promising treatment option for cancer. However, its utility may be limited due to expression in off-target cells. Cancer-specific promoters such as telomerase reverse transcriptase (TERT), survivin, and chemokine receptor 4 (CXCR4) have enhanced activity in a variety of human and murine cancers, however, little has been published regarding these promoters in dogs. Given the utility of canine cancer models, the activity of these promoters along with adenoviral E2F enhanced E1a promoter (EEE) was evaluated in a variety of canine tumors, both from the endogenous gene and from exogenously administered constructs. Endogenous expression levels were measured for cTERT, cSurvivin, and cCXCR4 and were low for all three, with some non-malignant and some tumor cell lines and tissues expressing the gene. Expression levels from exogenously supplied promoters were measured by both the number of cells expressing the construct and the intensity of expression in individual cells. Exogenously supplied promoters were active in more cells in all tumor lines than in normal cells, with the EEE promoter being most active, followed by cTERT. The intensity of expression varied more with cell type than with specific promoters. Ultimately, no single promoter was identified that would result in reliable expression, regardless of the tumor type. Thus, these findings imply that identification of a pan-cancer promoter may be difficult. In addition, this data raises the concern that endogenous expression analysis may not accurately predict exogenous promoter activity.

## Introduction

Gene therapy is a promising approach to treat different types of cancer. Cancer gene therapy aims to modify or kill cancerous cells [1], however, if used indiscriminately, may lead to serious side effects such as peripheral neuropathy and immunosuppression. This issue can be resolved by cancer-specific conditional gene expression to enhance robust therapeutic outcomes with relatively minimal side-effects [2, 3]. One such strategy is to employ tumor-upregulated or tissue-specific promoters to express therapeutic transgenes [4].

Promoters that are broadly upregulated across a variety of cancers with low expression levels in normal cells can serve as excellent candidates for driving therapeutic genes in cancer gene therapy. Examples include prostate–specific antigen (PSA) [5, 6], tyrosinase-related protein 1 (TRP-1), melanoma inhibitory activity (MIA), [7], and hepatocyte specific alpha-fetoprotein (AFP) [8–10]. We have selected three such upregulated promoters to study; survivin, chemokine receptor 4 (CXCR4) and telomerase reverse transcriptase (TERT).

Survivin is a bi-functional protein that promotes cell growth by inhibiting apoptosis. It is overexpressed in many cancers including breast [11], esophagus [12], lung [13], lymphoma [14], and others [15–17]. CXCR4 is a chemokine receptor that is expressed on most hematopoietic cells [18]. CXCR4 binding to CXCL12 ligand promotes gene transcription, chemotaxis, cell survival, proliferation, organ development, inflammation and immune surveillance of cells [19–21]. CXCR4 is also overexpressed in many cancers [22–24]. Telomerase reverse transcriptase (TERT) is an integral part of the telomerase enzyme complex. TERT restricts cell growth arrest and empowers the cells to undergo self-renewal [25–27]. TERT is highly upregulated in embryonic stem cells, progressively dividing cells, and cancer cells [28]. Likewise, TERT is overexpressed in many malignant diseases including lung cancer, gastric melanoma, prostate cancer, breast cancer, and various hematopoietic malignancies. [29–31].

Dogs are an outstanding translational animal cancer model for humans because they share the same environment, develop spontaneous cancers, and have similar genetic alterations and mechanisms to humans [32, 33]. Dogs are relatively outbred as compared to laboratory rodents (although purebred dogs present unique opportunities to study predisposition to certain cancer types) and represent an intermediate size that allows an approximation of the dose and scale that is required to successfully treat people [34–37]. While several studies reported successful utilization of TERT, survivin, and CXCR4 for transcriptional targeting in human cancers, none of these promoters have been investigated for their activity in canine tumors [38–43]. The goal of this study was to measure the activity of these promoters in a panel of canine tumors. In addition to these endogenous promoters, we also utilized a modified version of the canine adenoviral E1A promoter, containing four E2F binding sites and one Sp-I site (E2F enhanced E1A promoter, or EEE), which has previously been shown to be upregulated in activity in tumor cells [44].

## Materials and methods

### Cell culture

Canine melanoma (CML7, CML10), canine mammary tumor (CMT12, CMT28), canine histiocytoma (DH82), fetal dog kidney (FDK), Madin-Darby canine kidney cells (MDCK), and primary normal canine fibroblast (NCF) (**Table 1**) cells were cultured in DMEM (Dulbecco’s Modified Eagle's Medium, Corning) with penicillin (100 IU/ml, Corning), streptomycin (100 ug/ml, Corning), amphotericin B (0.5ug/ml, Corning), and 10% FBS (fetal bovine serum, Sigma). (NCF, CML7, CML10, CMT28 and DH82 were a gift from Dr. Richard C. Bird, College of Veterinary Medicine, Auburn University. MDCK and FDK were obtained from the American Type Culture Collection). Canine B cell lymphoma line 17–71 [45] (gift from Dr. Steven Suter, North Carolina State University) and peripheral T cell lymphoma line OSW [46] (gift from Dr. William C. Kisseberth, The Ohio State University) (**Table 1**) were cultured in RPMI (Roswell Park Memorial Institute medium, Corning) with penicillin (100 IU/ml, Corning), streptomycin (100 ug/ml, Corning), amphotericin B (0.5ug/ml, Corning) and 10% FBS (Sigma). All cells were grown at 37°C and 5% CO_2_ and were validated to be of canine origin by species-specific PCR [47]. All cell lines were observed for expected phenotypic appearance and verified as mycoplasma free (Universal Mycoplasma Detection Kit, ATCC).

**Table 1:** Cell lines.

Cell Name	Status	Description	Type
**NCF**	Adherent	Normal Canine Fibroblast	Primary
**PBMC**	Suspended	Peripheral Blood Mononuclear Cells	Primary
**MDCK**	Adherent	Madin-Darby Canine Kidney Cell lines	Cell line
**FDK**	Adherent	Fetal Dog Kidney	Cell line
**CML7**	Adherent	Canine Melanoma Cell line 7	Cell line
**CML10**	Adherent	Canine Melanoma Cell line 10	Cell line
**CMT28**	Adherent	Canine Mammary Tumor cell line 28	Cell line
**DH82**	Adherent	Canine Histiocytic Cell line 82	Cell line
**OSW**	Suspended	Canine Lymphoma Cell line	Cell line
**17–71**	Suspended	Canine Lymphoma Cell Line	Cell line

### Isolation of peripheral blood mononuclear cells (PBMCs)

PBMCs were isolated using SepMate™-50 tubes (Stem cell Technologies). 10 ml of blood was drawn from the cephalic vein of a normal adult female beagle dog into EDTA tubes (Tyco) and diluted with an equal amount of phosphate buffered saline (PBS, Corning) containing 2% FBS. The mixture was added to SepMate™-50 tubes containing Histopaque 1077 (Sigma). The mixture was centrifuged at 1200g for 10 min at room temperature. The supernatant was transferred to a 15ml conical tube, washed twice with PBS containing 2% FBS, and centrifuged at 300g for 8 min at room temperature. The supernatant was removed and the PBMC pellet was recovered for total RNA isolation. All animal work was performed in accordance with the Guide for the Care and Use of Laboratory Animals (National Institutes of Health) and was approved by the Auburn University Institutional Animal Care and Use Committee.

### Total RNA isolation, primer design and quantitative RT-PCR

Total RNA was isolated from cell lines, primary cells, normal canine tissues (lung, liver, heart, pancreas and kidney), and primary lymphoma tissues from client-owned dogs presenting with clinical lymphoma using Tri-reagent (Molecular Research Center, Inc.) (**Table 2 and S1 File**).

**Table 2:** Primary lymphoma tissues.

Cell Name	Status	Description	Type
**NCF**	Adherent	Normal Canine Fibroblast	Primary
**PBMC**	Suspended	Peripheral Blood Mononuclear Cells	Primary
**MDCK**	Adherent	Madin-Darby Canine Kidney Cell lines	Cell line
**FDK**	Adherent	Fetal Dog Kidney	Cell line
**CML7**	Adherent	Canine Melanoma Cell line 7	Cell line
**CML10**	Adherent	Canine Melanoma Cell line 10	Cell line
**CMT28**	Adherent	Canine Mammary Tumor cell line 28	Cell line
**DH82**	Adherent	Canine Histiocytic Cell line 82	Cell line
**OSW**	Suspended	Canine Lymphoma Cell line	Cell line
**17–71**	Suspended	Canine Lymphoma Cell Line	Cell line

All cells and frozen tissue samples (40-80mg) were homogenized in 1 ml Tri-reagent. RNA was isolated from the aqueous phase by isopropanol precipitation. RNA concentration was determined by absorbance at 260 nm. The mRNA expression from canine survivin, CXCR4, TERT, and beta actin (cSurvivin, cCXCR4, cTERT) was measured by quantitative reverse transcriptase PCR (Q-RT-PCR). The cDNA from 1ug of RNA was synthesized using an iScript cDNA synthesis kit (Bio-Rad, Catalog # 1708891). One tenth of the RT reaction volume was used for Q-RT-PCR assays using SSO-Advanced Universal syber green supermix (Bio-Rad) with 0.5uM forward and reverse primers in a volume of 20ul (**Table 3**). Thermocycling conditions were: 3 min at 95°C, then 40 cycles of 95^0^ C for 30 sec and 57°C for 30 sec, on a Bio-Rad iCycler iQ Multicolor Real-Time PCR Detection System. The mRNA expression was analyzed by the ΔCt method using beta-actin as the comparator. Primer specificity was validated by sequencing the PCR product (Eurofins MWG Operon).

**Table 3:** Quantitative reverse transcription PCR (Q-RT-PCR) primers.

Gene	Primer (5^/^-3^/^)	Amplicon size (bp)	GeneBank Accession No.
**β-actin Forward**	ACGGGCAGGTCATCACTATT	220	NM_001195845.1
**β-actin Reverse**	ATCTCCTTCTGAATCCTGTCA		
**cSurvivin Forward**	AAGAACTGGCCGTTCCTGGAGG	149	AY741504.1
**cSurvivin Reverse**	TCATCATCTGGCTCCCAGCCTTC		
**cCXCR4 Forward**	GCGGGCGAGCGGTTACCA	167	NM_001048026.1
**cCXCR4 Reverse**	GAAGATGATGGAGTAGACTGTGGGCAG		
**cTERT Forward**	GTTCATCTCCCTGGGAAAGCACG	172	NM_001031630.1
**cTERT Reverse**	GCCCATCAACCAGCACAAGGAAC		

Primers were designed using NCBI Primer blast. Primers are based on canine sequences.

### Enhanced green fluorescent protein (eGFP) reporter plasmid constructs

**Fig 1:**
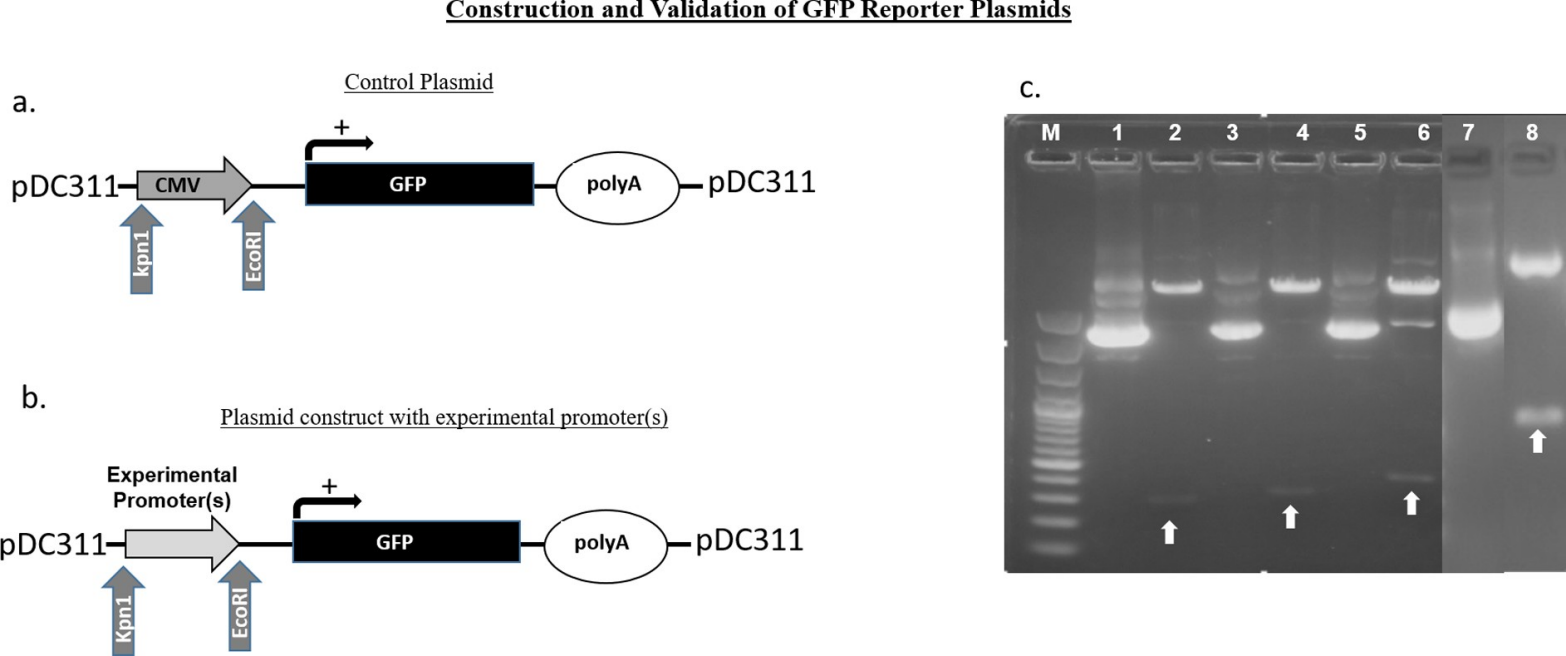
Construction of GFP reporter constructs driven by either (A) CMV promoter or (B) Experimental promoters. (C) Kpn1 and EcoRI mediated restriction digestion of experimental promoters containing plasmid constructs. M = 100bp ladder; 1 = pDC311-cSurvivin-GFP undigested plasmid (UDP); 2 = Kpn1 and EcoR1 double digested (DD) clone for cSurvivin-GFP construct; 3 = pDC311-cCXCR4-GFP UDP; 4 = Kpn1 and EcoR1 DD cCCXCR4-GFP construct; 5 = pDC311-cTERT-GFP UDP; 6 = Kpn1 and EcoR1 DD cTERT-GFP construct; 7 = pDC311-EEE-GFP UDP; 8 = EcoR1 and Kpn1 DD EEE-GFP construct. CMV–cytomegalovirus immediate early promoter, GFP–green fluorescent protein coding sequence, polyA–poly adenylation signal. White arrows indicate the location of the promoter fragment from the digested plasmid.

Reporter plasmids were constructed using pDC311CMV-eGFP plasmid (gift from Dr. Yadvinder Ahi, NIH) (**Fig 1A**). Canine Survivin, CXCR4 and TERT promoter sequences were predicted from the canine genome using NCBI Blast (**Table 4**). The recombinant expression vectors pDC311-cSurvivin-eGFP, pDC311-cCXCR4-eGFP, and pDC311-cTERT-eGFP were commercially synthesized (Gene Script Inc.) (**Fig 1B**). The EEE promoter sequence, with four imperfect palindromic E2F-binding sites and one Sp-I-binding site, was amplified using primers containing Kpn1 and EcoR1 restriction sites from pICOCAV15 (a gift from Dr. Ramon Alemany) [48]. The PCR product was digested with Kpn1 and EcoR1 and cloned into Kpn1/EcoR1 double digested pDC311-CMV-eGFP, replacing the CMV promoter with EEE to create pDC311-EEE-eGFP (**Fig 1B**). Recombinant plasmid constructs were confirmed by restriction digestion (**Fig 1C**) and sequencing (Eurofins MWG Operon).

**Table 4:** Promoters.

Promoter	Length (bp)	Chromosomal location	NCBI Reference Sequence
**Canine Survivin**	261bp	6	XM_022422541.1
**Canine CXCR4**	296bp	Unknown	Unknown
**Canine TERT**	379bp	34	AY563633.1
**E2F Modified E1A promoter (EEE)**	468bp	Not applicable	MN304825.1

### Transfection and flow cytometry

Adherent cells were transfected with jet prime transfection reagent (Polypus transfection, catalog# 114–07). Briefly, one day before transfection, 8 x 10^5^ adherent cells were seeded in a 24 well plate. On the day of transfection, 0.5ug of DNA was mixed with 50ul of jet prime buffer, and added to 1ul of jet prime reagent and incubated for 10 min. The transfection mix was then added dropwise to the medium in each well and incubated at 37°C for 48hr. Suspended cells were transfected via electroporation using the Neon Transfection System (Thermo Fisher Scientific, catalog # MPK1096). Briefly, the cells were washed with PBS and resuspended in resuspension buffer R at a final density of 2 × 10^7^ cells/ml. Then, the cells were mixed with 1ug DNA/ 2× 10^5^ cells and electroporated using 10ul Neon tips with the following conditions: pulse voltage: 1850, pulse width: 20, and pulse no: 1. Following electroporation, the cells were immediately put on ice for 15 min. The cells were then placed in 500ul of prewarmed serum containing antibiotic-free media and incubated for 48hr.

After two days, transfected adherent cells were harvested by trypsinization and electroporated cells were harvested by centrifugation. Cells were washed twice with PBS. The cells were resuspended in PBS and 0.1% BSA and analyzed for GFP expression by flow cytometry (Accuri C6).

### Statistical analysis

Promoter activity was compared using a two tailed student T test at the 95% confidence level by using Prism statistical software (GraphPad, San Diego, CA).

### Cell line validation statement

All cell lines were validated to be of canine origin by species specific PCR [47].

## Results

The activity of cSurvivin, cCXCR4, cTERT, and EEE was evaluated in canine cells to determine whether their expression was upregulated in canine tumors, based on upregulated expression in human tumors [16, 17, 31, 49–52]. Both endogenous and exogenous promoter activity was analyzed, the former by Q-RT-PCR and the latter by reporter gene assays.

### Analysis of endogenous promoter activity

**Fig 2:**
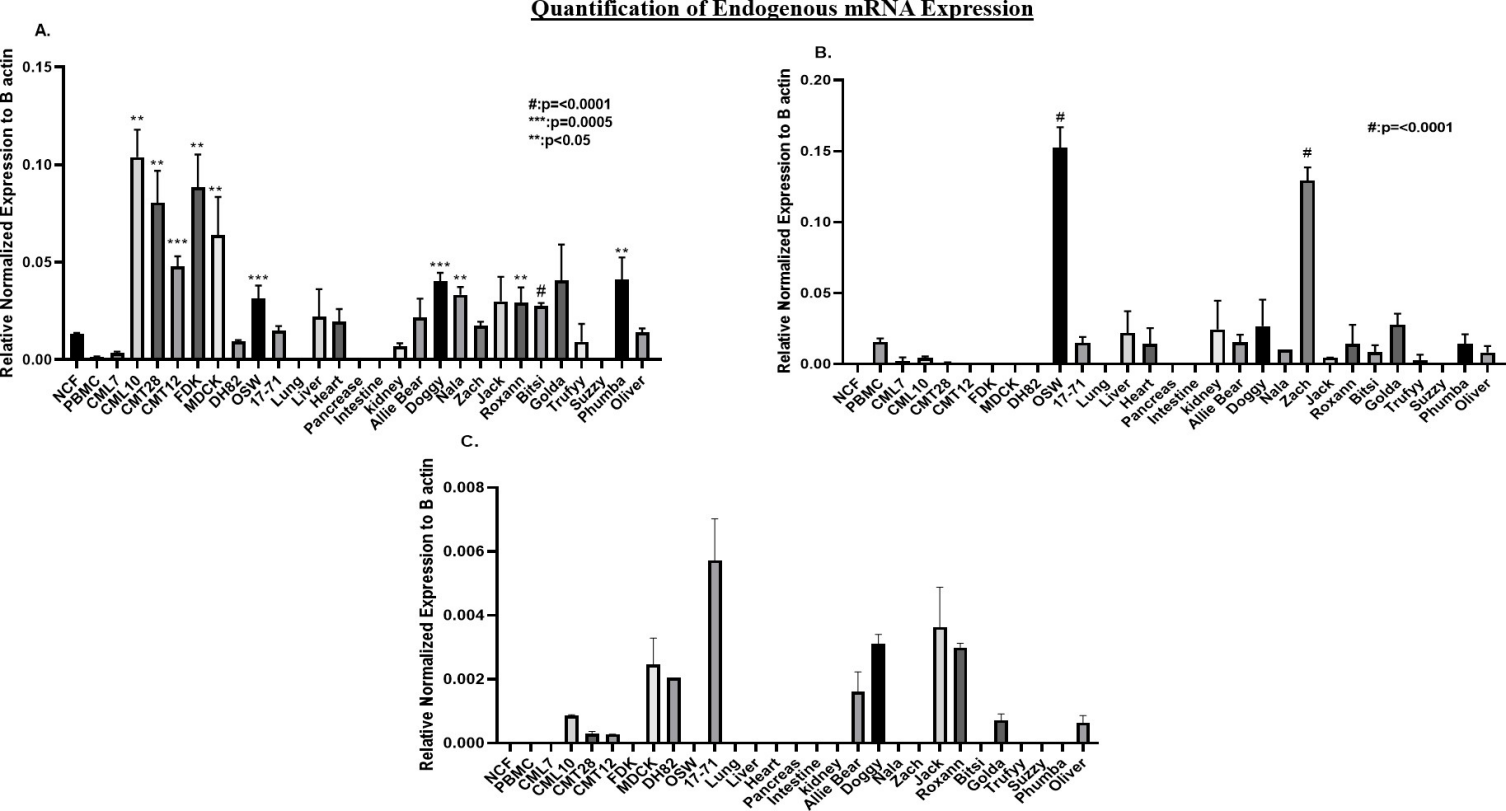
Quantification of endogenous mRNA expression of (A) cSurvivin, (B) cCXCR4 and (C) cTERT in normal canine cell lines /tissues and canine tumor cell lines/tissues using quantitative RT-PCR. Gene expression was normalized to β-actin (ΔCt method). Bar graphs are representative of mean and SEM of three independent experiments. Two tailed Student’s T test was conducted by using GraphPad prism statistical software. P-values represent comparisons with (A) NCF and (B) PBMC. No normal cells expressed cTERT and therefore statistical significance could not be calculated for Fig 2C.

Endogenous expression of cSurvivin, cCXCR4, and cTERT was analyzed by Q-RT-PCR in canine primary cells (NCF, PBMCs), cell lines (FDK, MDCK), cancer cell lines (CMT12, CMT28, CML7, CML10, DH82, 17–71, OSW), normal tissues (lung, liver, heart, pancreas, kidney, intestine), and primary tumor tissues (1 T cell lymphoma, 11 B cell lymphomas) **(Tables 1 and 2)**. In order to compensate for the different efficiencies in RNA isolation among cultured cells and tissues, mRNA expression for each gene was normalized to beta-actin expression (ΔCT) in each cell or tissue type (**Fig 2**). Results showed that beta-actin levels varied depending on the source of RNA (cells/cell lines/tissues), and relatively consistent beta-actin levels were observed within the tested cells/cell lines and within the tissue samples (**S2 File**). While it is possible that actual beta-actin expression levels may vary between cultured cells and tissue samples given the consistency within each group, it is more likely that, these variations were the result of different efficiencies in RNA isolation between cultured cells and tissue samples. Such variation in RNA recovery should affect both beta-actin and the genes of interest proportionally, and thus justifies the utilization of beta-actin was to normalize the mRNA expression for the tested promoters.

cSurvivin expression was not detectable in PBMCs, lung, pancreas, intestine, and one primary lymphoma (Suzzy). Low levels of expression were observed in NCF, kidney, heart, liver, CML7, 17–71, DH82, and four primary lymphomas (Zach, Oliver, Allie Bear, Truffy), ranging from 0.1% to 2.2% of beta-actin. Moderate levels of expression were observed in OSW and seven primary lymphoma samples (Phumba, Golda, Doggy, Bitsi, Jack, Roxann, Nala), ranging from 2.75% to 4.1%. The highest levels of expression were observed in CML10, CMT28, CMT12 and MDCK, varying from 4.7% to 10.4% (**Fig 2A**).

cCXCR4 mRNA expression was undetectable in NCF, FDK, MDCK, DH82, normal lungs, liver, pancreas, and intestine. Low expression, ranging from barely detectable (0.05%) to 3.1% was seen in PBMCs, heart, kidney, CML7, CML10, CMT12, CMT28, and 17–71, as well as in lymphoma tissues from Doggy, Golda, Roxann, Allie bear, Phumba, Jack, Bitsi, Truffy, and Oliver, with the highest expression in this group reaching 3.1%. OSW (15%) and lymphoma tissue from Zach (12.9%) showed clearly elevated expression levels (**Fig 2B**).

cTERT levels were 1 to 2 orders of magnitude lower than either cSurvivin or cCXCR4 in all tissues studied. cTERT was undetectable in most of the normal cells/tissues including NCF, PBMCs, lungs, liver, heart, pancreas, intestine and kidney. It was also undetectable in some cancer cell lines and primary lymphoma tissues including CML7, FDK, OSW, Nala, Zach, Truffy, Suzy, Bitsi, and Phumba. Low levels of expression were seen in CML10, CMT28, CMT12, and MDCK, and primary lymphoma tissue samples from Allie bear, Doggy, Jack, Roxann, and Golda. The highest expression of cTERT was seen in 17–71 (0.5%) (**Fig 2C**).

### Analysis of exogenous promoter activity in primary cancer cells and cell lines

**Fig 3:**
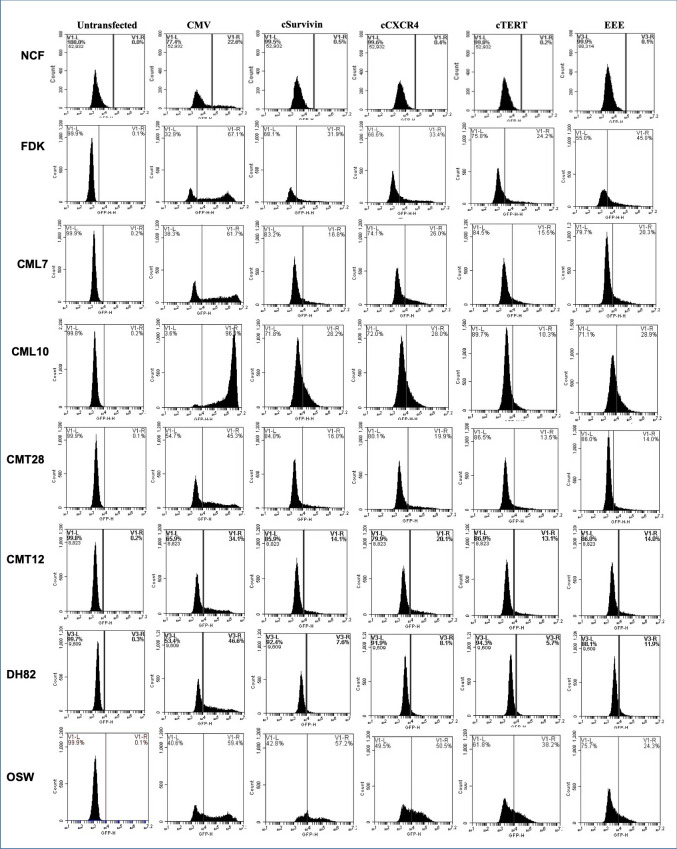
Flow cytometric analysis of exogenous promoter activity.

The activity of exogenously provided cSurvivin, cCXCR4, cTERT, and EEE promoters was evaluated in normal and cancer cells. Reporter plasmids expressing GFP, driven by CMV (positive control), cSurvivin, cCXCR4, cTERT, or EEE promoters were transfected into NCF, FDK, CML7, CML10, CMT12, CMT28, DH82, and OSW cells. GFP expression was quantified by flow cytometry 48hrs after transfection. (**Fig 3**). Flow cytometry provided results for both the number of cells expressing the experimental promoter and the intensity of the expression within a given cell. These parameters were analyzed independently.

#### Analysis of number of cells expressing the experimental promoter

**Fig 4:**
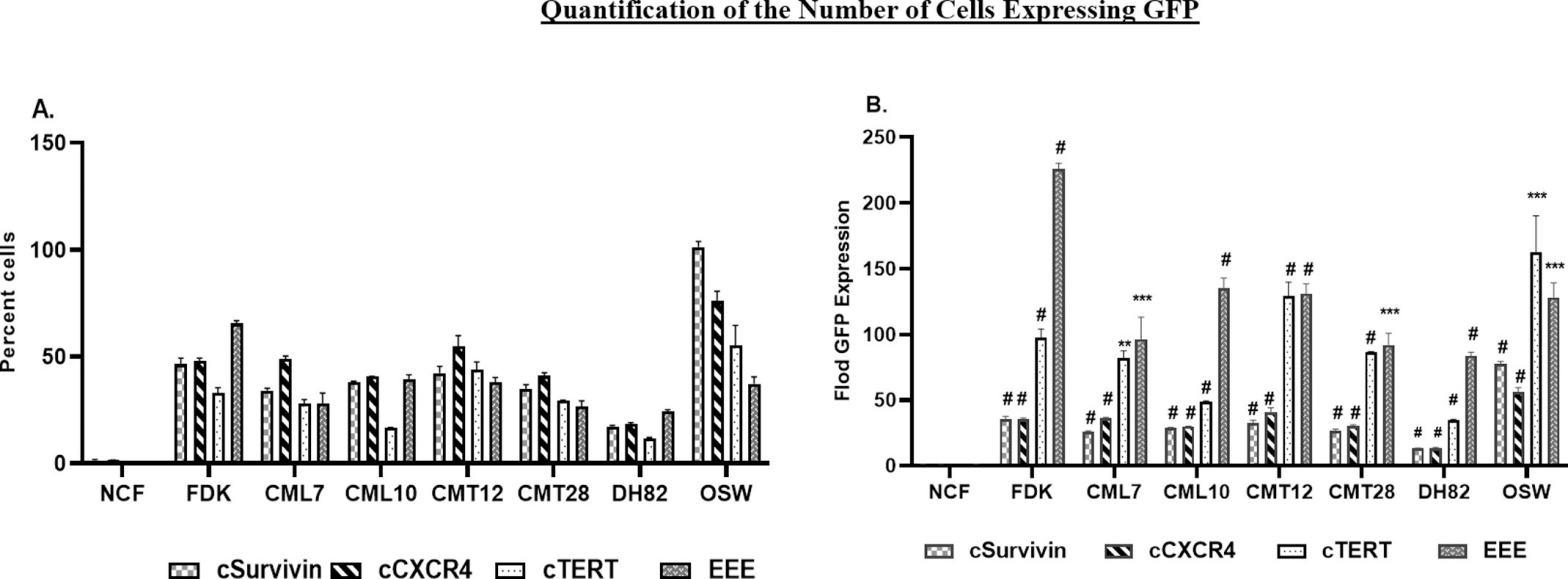
Quantification of the number of cells expressing GFP normalized to either A) CMV-GFP expression or B) NCF to compare the significance of the observed variation in promoter activity. Bar graphs are representative of mean and SEM of three independent experiments. Two tailed Student’s T test was conducted by using GraphPad prism statistical software. P-values represent comparisons with NCF (B) and are represented as # (p<0.001), *** (p<0.005), and ** (p< 0.05).

In order to analyze differences in the number of cells expressing the GFP reporter from the experimental promoter, it was necessary to control for transfection efficiency. Given the high level and ubiquitous nature of CMV immediate early promoter expression, the expression of GFP from this promoter was used to measure transfection efficiency [53, 54]. The number of cells expressing GFP from the experimental promoter was then normalized to the number of cells expressing CMV driven GFP in that cell line (**Fig 4A and Table 5**). In NCF, all four promoters showed the fewest cells expressing GFP, relative to CMV. EEE activity was lowest at 0.29%, cTERT was slightly higher at 0.34%, and cSurvivin and cCXCR4 were the highest at 1.3% and 1.4% respectively. The number of cells showing promoter activity was notably increased in the remaining cells lines. The lowest expression was cTERT in DH82 cells at 11.7%. The highest percentage of cells expressing GFP occurred with the cSurvivin promoter in OSW cells (101.2%). The second highest percentage was cCXCR4 in OSW cells (75.9%). The third highest expression level was the EEE promoter in FDK cells (65.1%). The fourth highest level of expression was the cTERT promoter in OSW cells (55.1%) **(Fig 4A and Table 5)**.

**Table 5:** Percent cells expressing GFP.

	cSurvivin	cCXCR4	cTERT	EEE
**NCF**	1.3	1.4	0.34	0.29
**FDK**	46.3	48.0	33.1	65.1
**CML7**	34.3	48.5	27.8	28.2
**CML10**	38.0	40.6	16.6	39.2
**CMT12**	43.8	57.9	44.4	38.2
**CMT28**	34.7	40.9	29.3	26.8
**DH82**	17.2	18.4	11.7	24.3
**OSW**	101.2	75.9	55.1	37.3

The percent of cells expressing GFP for each cell line are ranked by color as follows: Red > Yellow >Dark Green > Light Green. Color code changed if the difference is >0.3

In general, OSW cells had provided the highest or nearly the highest number of cells expressing GFP from the experimental promoters, while DH82 cells had provided the lowest number of cells expressing GFP from the experimental promoters when tumor lines were compared. Within each cell type, the promoters showed different relative activities. For example, promoter activity in OSW, as ranked from highest to lowest was: cSurvivin, cCXCR4, cTERT, and EEE; whereas CML10 activities were ordered: cCXCR4, EEE, cSurvivin, cTERT. **Table 5**, where the order of activity of each promoter is color-coded within each cell line, demonstrates that cCXCR4 has the highest activity in 5 cell lines and second highest in three others, while cSurvivin has the highest activity in two cell lines and the second highest activity in two other lines **(Fig 4A and Table 5)**.

To compare the activities (fold difference) of promoters between cancer cell lines and normal cells (NCF), an important measure of the specificity of the promoter for cancer cell gene expression, the CMV normalized expression values were normalized to values for NCF (**Fig 4B and Table 6**). Among all of the tested promoters, EEE showed the highest relative activity, ranging from 83.9 to 224.9 times higher in cancer cell lines than in NCF. The EEE promoter generated the highest amount of relative expression in every cell line except OSW, where it was the second most active, following cTERT. cTERT was second most active in all of the cell lines except OSW. cSurvivin and cCXCR4 expression levels were very similar, with cSurvivin slightly higher than cCXCR4 in 6 lines and lower than cCXC4 in one line (OSW). However, expression levels of both of these promoters were well below that of EEE and cTERT.

**Table 6:** Fold GFP expression.

	cSurvivin	p-Value	cCXCR4	p-Value	cTERT	p-Value	EEE	p-Value
**NCF**	1.0		1.0		1.0		1.0	
**FDK**	35.4	p = 0.0001	35.5	p<0.0001	97.5	p = 0.0001	225	p<0.0001
**CML7**	26.2	p<0.0001	35.8	p<0.0001	82.0	p = 0.0001	97.4	p = 0.0046
**CML10**	26.9	p<0.0001	30.0	p<0.0001	48.9	p<0.0001	135.2	p<0.0001
**CMT12**	33.5	p = 0.0002	42.8	p = 0.0004	130.9	p = 0.0003	132.0	p<0.0001
**CMT28**	26.5	p = 0.0001	30.2	p<0.0001	86.5	p<0.0001	92.5	p = 0.0005
**DH82**	13.1	p<0.0001	13.6	p<0.0001	34.5	p<0.0001	83.9	p<0.0001
**OSW**	77.4	p<0.0001	56.1	p<0.0001	162.6	p = 0.004	128.7	p = 0.0003

The percent of cells expressing GFP for each cell line are ranked by color as follows: Red > Yellow >Dark Green > Light Green. Color code changed if the difference is >0.3.

#### Analysis of promoter strength by measuring mean fluorescence intensity (MFI)

In order to gather quantitative information regarding promoter strength, we analyzed MFI from flow cytometry to estimate the amount of GFP produced in each transfected cell by each promoter.

**Fig 5:**
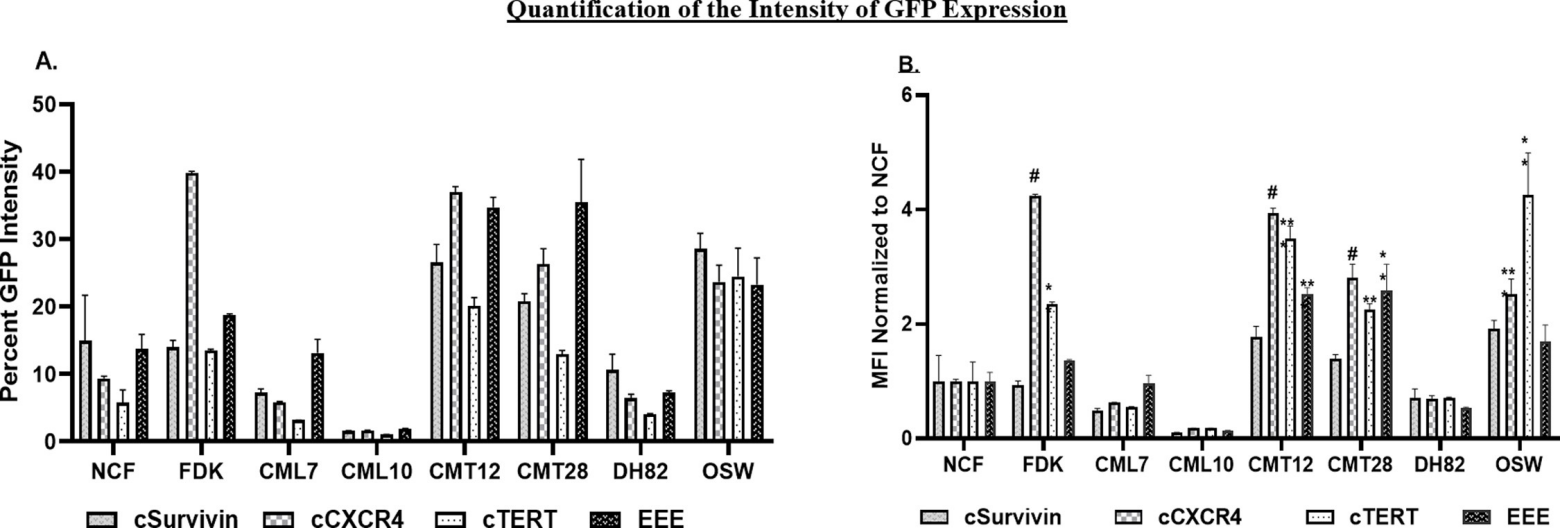
Quantification of mean fluorescent intensity (MFI) normalized to either A) CMV-GFP expression or B) NCF to compare the significance of the observed variation in promoter the strength. Bar graphs are representative of mean and SEM of three independent experiments. Two tailed Student’s T test was conducted by using GraphPad prism statistical software. P-values represent comparisons with NCF (B) and are represented as # (p≤0.0001), *** (p<0.005), and ** (p< 0.05).

When the data from flow cytometry is examined (**Fig 3)**, the maximum fluorescence intensity with the CMV promoter was fairly uniform in all cell types, however, the MFI varied between the cells/cell lines. Thus, while each cell type is capable of providing the same maximum intensity, the MFI varies based on the transfection efficiency. Low transfection efficiency results in less plasmid copy number within the target cells leading to variability in the MFI as observed for the tested cell lines. In order to compensate for the observed variability resulting from variation in transfection efficiency, the MFI from each promoter was normalized to MFI for CMV-GFP expression in that cell line to directly compare the promoter strength within cell lines (**Fig 5A**).

None of the promoters achieved levels of GFP expression seen with CMV. Significant variation in promoter strength was observed. Higher levels of expression for all 4 promoters were seen in NCF than in CML7, CML10, and DH82. CML10 showed the lowest fluorescent intensities, ranging from 1.0% to 1.9%. CMT12, CMT28 and OSW showed the highest fluorescent intensities with a range of 13.0% to 37.0%. cSurvivin showed a moderate level of strength in NCF, DH82, FDK, and CML7 ranging from 7.3% to 14.9% MFI (**Fig 5A and Table 7**).

**Table 7:** Promoter strength measured by MFI.

	cSurvivin	cCXCR4	cTERT	EEE
**NCF**	**14.9**	**9.4**	**5.8**	**13.7**
**FDK**	**14.0**	**39.8**	**13.5**	**18.8**
**CML7**	**7.3**	**5.8**	**3.2**	**13.1**
**CML10**	**1.6**	**1.7**	**1.0**	**1.9**
**CMT12**	**26.6**	**37.0**	**20.1**	**34.7**
**CMT28**	**20.8**	**26.4**	**13.0**	**35.5**
**DH82**	**10.6**	**6.5**	**4.1**	**7.3**
**OSW**	**28.6**	**23.7**	**24.5**	**23.3**

The promoter strength for each cell line is ranked by color as follows: Red > Yellow >Dark Green > Light Green. Color code changed if the difference is >0.3.

cCXCR4 was the most active promoter, displaying 39.8% of the activity of CMV in FDK and slightly lower levels in CMT12, CMT28, and OSW (23.7% to 37%). cCXCR4 showed moderate strength in NCF at 9.4%. In contrast, cCXCR4 showed low strength in DH82, CML7, and CML10 (1.7% to 6.5% MFI). cTERT showed low strength in NCF, DH82, CML7, and CML10 (1.0% to 5.8%) and significantly higher levels of strength in FDK, CMT12, CMT28, and OSW (13.5% to 24.5%). EEE showed moderate strength in NCF, FDK, and CML7 (13.1% to 18.8%), and a high level of strength in CMT12, CMT28 and OSW (34.7%, 35.5%, and 23.3%). The EEE promoter showed low levels of strength in CML10 and DH82 (1.9% and 7.3%) (**Table 7**).

When the order of activity of each promoter was color coded within each cell line (**Table 7**), several generalities about expression were seen. The cTERT promoter is almost uniformly the weakest, placing last in every cell line but one, OSW, where it was second strongest. The remaining 3 promoters are each strongest in 2 or 3 cells lines, with a slight overall advantage in strength going to the EEE promoter, which was strongest in 3 cells lines (CML7, CML10, CMT28) and second strongest in an additional 4 cell lines (NCF, FDK, CMT12, DH82).

In order to compare the strength of the tested promoter in cancer cells to the strength of the same promoter in NCF, the normalized MFI for each promoter in each cell line was normalized to NCF. This comparison allows the relative expression in cancer versus normal to be determined, giving an indication of the potential specificity of expression. These values are relative and not absolute; thus it is understood that a promoter expressing at very high levels in a tumor cell, with moderate background in normal cells, may have a lower relative expression value than a promoter expressing at moderate levels in tumor and extremely low levels in normal cells. Three cell lines, CML7, CML10, and DH82, failed to achieve the same level of expression as NCF with any of the promoters. The remaining cell lines showed increased expression for most or all of the tested promoters, but the pattern was highly variable (**Fig 5B and Table 8**).

**Table 8:** MFI normalized to NCF.

	cSurvivin	p-Value	cCXCR4	p-Value	cTERT	p-Value	EEE	p-Value
**NCF**	1.0		1.0		1.0		1.0	
**FDK**	0.936	p = 0.895	4.24	p<0.0001	2.35	p = 0.015	1.37	p = 0.080
**CML7**	0.486	p = 0.321	0.622	p = 0.0004*	0.554	p = 0.249	0.957	p = 0.850
**CML10**	0.105	p = 0.118	0.180	p<0.0001*	0.180	p = 0.068	0.140	p = 0.005*
**CMT12**	1.78	p = 0.186	3.94	p<0.0001	3.50	p = 0.003	2.53	p = 0.001
**CMT28**	1.39	p = 0.441	2.81	p = 0.0017	2.26	p = 0.022	2.58	p = 0.031
**DH82**	0.709	p = 0.577	0.688	p = 0.011*	0.714	p = 0.437	0.529	p = 0.040*
**OSW**	1.91	p = 0.118	2.52	p = 0.004	4.26	p = 0.015	1.69	p = 0.103

The MFI normalized to NCF for each cell line is ranked by color as follows: Red > Yellow >Dark Green > Light Green

*: strength of promoter in this cell line is significantly less than that of NCF. Color code changed if the difference is >0.3.

cSurvivin failed to show a statistically significant increase in the intensity of GFP expression compared to NCF in any of the cell lines tested. cCXCR4 showed the highest increase of 4.24-fold in FDK and also showed significant increases over NCF in CMT12, CMT28, and OSW [3.94 (p<0.0001), 2.81 (p<0.0001), 2.52 (p<0.0001) respectively]. cTERT showed significantly enhanced GFP expression in OSW and CMT12 of 4.26 (p<0.0001,) and 3.50 (p = 0.0004), respectively. EEE showed statistically significant increased GFP expression in two cell lines, CMT12 and CMT28, with 2.53 (p = 0.0020) and 2.58 (p = 0.0014) times higher MFI than NCF, respectively. The remaining cell lines, OSW, FDK, CML7, DH82 and CML10, failed to show any significant increase in expression with EEE over that seen in NCF.

## Discussion

Utilization of transcriptional targeting could improve the safety and efficacy of cancer gene therapy approaches. The identification of suitable tumor-upregulated promoters is a prerequisite for this approach. Although several tumor-upregulated promoters have been utilized for human cancer gene therapy, none have been tested in canine tumors [40, 41, 43, 55–57]. Given that many canine tumors are excellent models of their human counterparts, evaluation of these promoters in canine cells would provide valuable comparative information. In the present study, we selected the 5^’^ upstream region of the cSurvivin, cCXCR4 and cTERT genes, based on their tumor-upregulated activity in human cancer. The endogenous activity of these promoters was assessed by Q-RT-PCR. The exogenous activity of these promoters, as well as a modified CAV2 E1A promoter (EEE) was evaluated using a GFP reporter gene.

Endogenous mRNA expression provides an estimate of activity of promoters in cells. In this regard, we measured mRNA expression levels of cSurvivin, cCXCR4 and cTERT genes (**Fig 2**). All the promoters had very low levels of mRNA expression in normal cells and tissues. Among the tested genes, survivin showed the highest level of endogenous activity. Elevated survivin expression in human and murine melanoma, mammary cancer, and lymphoma have been previously reported [40, 41, 55, 58]. We have observed similar expression levels in canine cell lines. There was a moderate level of mRNA expression in one lymphoma line, and high levels of mRNA expression in one melanoma and two mammary tumor cell lines. Our normal tissue results are congruent with human and mouse data where low or no survivin transcript was observed in lung, liver, heart, pancreas and kidney etc. This is in contrast to a report of high levels of survivin mRNA in various normal dog tissues [59].

Elevated expression of CXCR4 has been reported in human mammary cancer, melanoma and B cell non-Hodgkin’s lymphoma (NHL) [60–63]. However, we found little or no expression of cCXCR4 mRNA in most of the normal and cancer samples, including a B cell lymphoma cell line (17–71). CXCR4 mRNA overexpression has been reported in T-cell NHLs [64, 65]. Likewise, high levels of CXCR4 mRNA expression were found in OSW and Zach, which are both T cell lymphomas, but no expression was seen in canine B cell lymphomas. cTERT showed the lowest level of endogenous mRNA expression in all of the cell lines and tissues, with maximal activity being one to two orders of magnitude lower than cSurvivin or CXCR4, indicating that TERT transcriptional activity is low in all canine tumors and tissues. Relative to NCF, cTERT expression increased in cell lines CML10, MDCK, DH82, and 17–71, as well as in primary lymphoma tissues from 6 of 12 dogs.

Based on these findings, cSurvivin gene expression was most consistently elevated in tumor samples, indicating that it might be a good choice for therapeutic applications. However, based on the endogenous expression data in normal cells and tissues, there could be significant off-target expression. The activity of endogenous cCXCR4 and cTERT appear to be limited to a few tumor types. The low level of endogenous expression of these genes relative to beta-actin also raises concern for their therapeutic utility. However, typical gene therapy applications rely on the use of exogenously provided promoter constructs. The alteration of chromosomal context combined with the selection of specific portions of the promoter might provide different expression profiles. For this reason, the three endogenous promoters, along with the modified CAV2 E1A promoter, EEE, were tested for their expression levels when provided exogenously.

The use of flow cytometry to assess exogenous expression allows parameters of expression to be evaluated, including the number of cells expressing the construct and the intensity of that expression in individual cells. The former indicates the breadth of expression while the latter indicates its strength. These experiments present a conundrum with respect to appropriate controls for transfection. Ideally the cells being tested would be co-transfected with a plasmid bearing another fluorescent reporter construct driven by ubiquitously active promoter such as SV40 or CMV. However, co-transfection of a CMV driven control plasmid along with the plasmids bearing the experimental promoters routinely and reliably generated lower expression from the experimental promoters than transfection of these plasmid alone. Thus, the co-transfection of the reporter plasmid was resulting in inhibition of the experimental promoter. This phenomenon of transcriptional interference has been noted by others [66–70]. Based on these findings, independent transfections were used for this study. Transfections were performed in triplicate and the tight statistical grouping of these triplicates indicates that little experimental variation in transfection efficiency occurred within a specific cell type or line. Thus, we strongly believe that, our reported promoter activity in cancer cells is the true reflection from the normalized fluorescence intensity rather than an artifact generated from variable transfection efficiencies.

The number of cells expressing the exogenous constructs was elevated for all the promoters in all cancer cell lines tested when compared to NCF (**Fig 4 and Table 5**). The cCXCR4 promoter was active in a higher percentage of cells in 4 cell lines, with the EEE promoter showing the highest number of cells expressing GFP in two lines and the cSurvivin promoter showing the highest number of cells expressing GFP in one line and NCF. When the intensity of expression was evaluated, expression levels appear to correlate more with cell lines than with specific promoters (**Fig 5 and Table 6)**. For example, expression of all four promoters was elevated above that of NCF in both mammary carcinoma lines (CMT12, CMT28) and a T-cell lymphoma (OSW), while the intensity of expression was at or below the level of NCF in both melanoma lines (CML7, CML10) and the histiocytoma line, DH82. In cells with elevated fluorescent intensity, EEE had the greatest intensity in 6 lines followed by cTERT, which was highest in 1 cell line (**Table 6)**. It must be remembered that when comparing the MFI, very small numbers of NCF are being compared to much larger numbers of the other cells.

The immortalized fetal dog kidney (FDK) was ostensibly included as a second normal control, however, FDK showed increased endogenous expression with the survivin promoter. When exogenous expression was examined in FDK, the number of cells expressing GFP was elevated with all 4 promoters. When the intensity of expression was examined, FDK showed a range of increased expression over NCF, from no increase with cSurvivin to a moderate increase with cTERT and EEE, to a more than 4-fold increase with cCXCR4. Immortalization of FDK with SV40 Large-T antigen has likely resulted in FDK more closely resembling tumor cells than normal fibroblasts. The fetal origin of FDK might also contribute to this phenotype

Comparison of the number of cells expressing the exogenous promoters to the level of expression with those promoters shows that these metrics are not well correlated across the panel of tumor cells tested. In some cases, such as CML7, CML10, and DH82, the tumor cells showed an increased number of cells expressing lower levels of GFP than are seen in normal fibroblasts. The low level of expression of the reporter in these tumor cells raises the concern that the use of these promoters might result in unacceptable side effects in normal cells. In contradistinction, in CMT12, CMT28, and OSW, both the number of cells and the intensity of fluorescence were elevated for all four promoters, indicating that more cells express these promoters and at a higher level than in normal canine fibroblasts, making these promoters potentially attractive for therapy in tumor cells of this type.

In this study, the mRNA level observed for endogenous expression differed from the high activity observed with exogenous expression from the same promoters. The data from the endogenous studies reflects the efficiency with which the chromosomal promoters mediate gene expression. Inside the cell, endogenous promoters maintain their own chromatin structure and are controlled by a complex network of regulatory elements such as transcription factors, activators and mediators. Regulation of mRNA levels in a cancer cell may be the outcome of multiple events such as mutation, chromatin remodeling, epigenetic modifications, and microRNA activity. In contrast, exogenously supplied promoters are putative promoter fragments cloned into a plasmid bearing reporter genes that lack the native chromatin structure and genomic context. As a consequence, mRNA and/or protein levels produced from the endogenous genes may not correlate with the exogenous activity derived from the same promoter in the same cell. In addition, positive feed-back loops may also play an important role during exogenous activity promoting new protein to act as an activator resulting in high activity of the promoter [71–74].

Our primary objective was to find a tumor upregulated promoter that could be used as a transcriptional targeting tool for all types of cancer. Our data indicates that none of the promoters tested completely satisfy this requirement. Endogenous expression from cTERT, cSurvivin, and cCXCR4 was low, and increased expression was only seen in some cell types. Exogenously provided promoters were ubiquitous in their expression in more tumor cells than normal cells, but expression levels varied, with some being expressed at much lower intensities than in normal cells. Thus, it is clear that, among the promoters and cells studied, there is no “magic bullet” promoter that can be used in all, or even most cancers. The use of specific promoters will require individualized testing in a precision medicine-based approach. Even that approach has difficulties, as these results indicate that precision approaches such as transcriptome sequencing may seriously underestimate both the promoters that might be active, if provided exogenously, and the level of expression that could be expected from those constructs.

## Supporting information

S1 Raw image Kpn1 and EcoRI mediated restriction digestion experimental promoters containing plasmid constructs A) Restriction digestion of cSurvivin-GFP, cCXCR4-GFP and cTERT-GFP construct; M = 100kb ladder; 1 = pDC311-cSurvivin-GFP undigested plasmid (UDP); 2 = Kpn1 and EcoR1 double digested (DD) clone for cSurvivin-GFP construct; 3 = pDC311-cCXCR4-GFP UDP; 4 = Kpn1 and EcoR1 DD cCCXCR4-GFP construct; 5 = pDC311-cTERT-GFP UDP; 6 = Kpn1 and EcoR1 DD cTERT-GFP construct; B) Restriction digestion of EEE-GFP construct. 1 = pDC311-EEE-GFP undigested plasmid (UDP); 2) Kpn1 singled digested EEE-GFP construct; 4) EcoRI singled digested EEE-GFP construct; 5 = Kpn1and EcoRI double digested EEE-GFP construct. CMV–cytomegalovirus immediate early promoter, GFP–green fluorescent protein coding sequence, polyA–poly adenylation signal. White arrows indicate the location of the promoter fragment from the digested plasmid.
(TIF)

S1 File Details about the dogs that provided the lymphoma samples used this study. (XLSX)

S2 File Dataset for endogenous promoter activity. (XLSX)

S3 File Dataset for exogenous promoter activity. (XLSX)
