# Supplementary figures and images for: Analysis of endogenous and exogenous tumor upregulated promoter expression in canine tumors
Source: PLoS One. 2020 Nov 9;15(11):e0240807. doi: 10.1371/journal.pone.0240807 (PMC7652315; doi:10.1371/journal.pone.0240807)

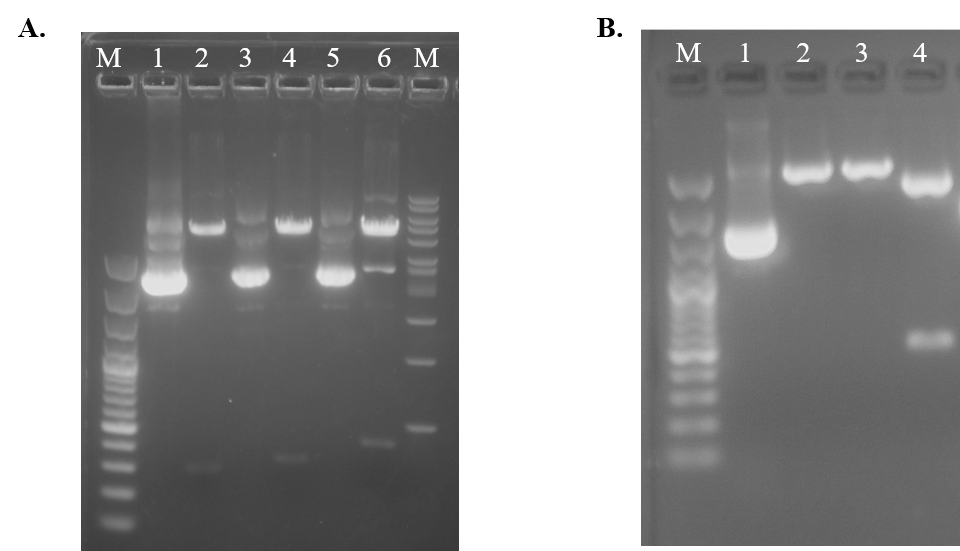

Supplement: S1 Raw image — Kpn1 and EcoRI mediated restriction digestion experimental promoters containing plasmid constructs A) Restriction digestion of cSurvivin-GFP, cCXCR4-GFP and cTERT-GFP construct; M = 100kb ladder; 1 = pDC311-cSurvivin-GFP undigested plasmid (UDP); 2 = Kpn1 and EcoR1 double digested (DD) clone for cSurvivin-GFP construct; 3 = pDC311-cCXCR4-GFP UDP; 4 = Kpn1 and EcoR1 DD cCCXCR4-GFP construct; 5 = pDC311-cTERT-GFP UDP; 6 = Kpn1 and EcoR1 DD cTERT-GFP construct; B) Restriction digestion of EEE-GFP construct. 1 = pDC311-EEE-GFP undigested plasmid (UDP); 2) Kpn1 singled digested EEE-GFP construct; 4) EcoRI singled digested EEE-GFP construct; 5 = Kpn1and EcoRI double digested EEE-GFP construct. CMV–cytomegalovirus immediate early promoter, GFP–green fluorescent protein coding sequence, polyA–poly adenylation signal. White arrows indicate the location of the promoter fragment from the digested plasmid. (TIF) [file pone.0240807.s001.tif]
